# Intraoperative Diagnosis of "Bronchus Suis", a Variant of Tracheal Bronchus

**DOI:** 10.7759/cureus.29498

**Published:** 2022-09-23

**Authors:** Didem Tan, Somdatta Gupta, Michael Block

**Affiliations:** 1 Department of Anesthesiology, Hackensack University Medical Center, Hackensack, USA

**Keywords:** pig bronchus, intraoperative hypoxemia, endotracheal intubation, bronchus suis, tracheal anomaly, tracheal bronchus

## Abstract

Bronchus suis, also known as tracheal bronchus, is a rare congenital anatomic variant in which an aberrant bronchus develops above the carina. Often asymptomatic, bronchus suis may be detected during imaging or manipulation of the airway. Here we describe a case where bronchus suis was visualized by fiberoptic bronchoscopy performed intraoperatively for the evaluation of desaturation and management of endotracheal secretions. It is important for anesthesiologists to consider the possibility of tracheal bronchus in patients with acute respiratory compromise.

## Introduction

"Bronchus suis" or "pig bronchus", a subtype of tracheal bronchus, is an airway anomaly in which a bronchus arises above the carina and branches into the entire upper lobe of the lung, most commonly on the right side [1-4]. When the tracheal bronchus pathology is severe, it is often associated with other congenital anomalies or involves narrowing/obstruction of the distal airway [1-5]. Neonates and children may present with moderate to life-threatening respiratory symptoms if the airway anomaly is severe [1-3]. In less severe cases, patients are typically diagnosed in adulthood and have minimal or mild symptoms [1-3]. The diagnosis may be incidental during imaging or may present in an unanticipated airway emergency [2,3]. Our patient was a previously undiagnosed adult who was intubated for an upper endoscopy and had progressively worsening intraoperative hypoxia during the case. Fiberoptic bronchoscopy performed to remove secretions revealed the opening of the right upper bronchus to be above the carina, supplying the upper lobe of the right lung. 

## Case presentation

A 27-year-old female with a past medical history of hypertension, diabetes mellitus, systemic lupus erythematosus, and end-stage renal disease on hemodialysis, was admitted for acute upper gastrointestinal bleeding. Upper and lower endoscopies were planned. The preoperative saturation of peripheral oxygen (SpO2) was 98% in room air. The patient underwent rapid sequence intubation with video laryngoscopy and the endotracheal tube (ETT) was secured at 20 cm at the lip. Intraoperatively, the patient had brief episodes of hypoxia (SpO2 85-90%), which temporarily improved with alveolar recruitment maneuvers. Following the reversal of neuromuscular blockade and emergence from inhalational anesthesia, the patient was noted to be breathing spontaneously more than 25 breaths per minute with low tidal volumes. As a result, the patient remained intubated. Shortly thereafter, the SpO2 rapidly decreased to 70%. The patient received manual ventilation and additional recruitment maneuvers with minimal improvement. Copious clear secretions were noted in the ETT that were not present initially. Fiberoptic bronchoscopy was performed to assess and suction the airway. The ETT was noted to be in the right main stem bronchus and withdrawn 2 cm. The right upper bronchus was seen to arise above the carina. All branches of the right main bronchus were visualized for verification and the ETT was re-secured at 18 cm at the lip, above the tracheal bronchus opening. Shortly thereafter, the patient's ventilation and oxygenation improved with manual ventilation. It was concluded that the inadequate ventilation of the right upper lobe due to tracheal bronchus, in addition to the left lung, might be the reason for the major drop in SpO2. The patient remained intubated and was transported for hemodialysis and further imaging (CT scan). The patient was extubated the following day with no further complications.

## Discussion

Tracheal bronchus is defined as a bronchial opening above the carina [1-5]. The most common location is between 2 cm and 6 cm above the carina [1-5]. This congenital anomaly is more commonly right-sided and occurs early in the embryonic stage of development [2,5]. The prevalence of tracheal bronchus is 0.1-3% [2,3].

**Figure 1:**
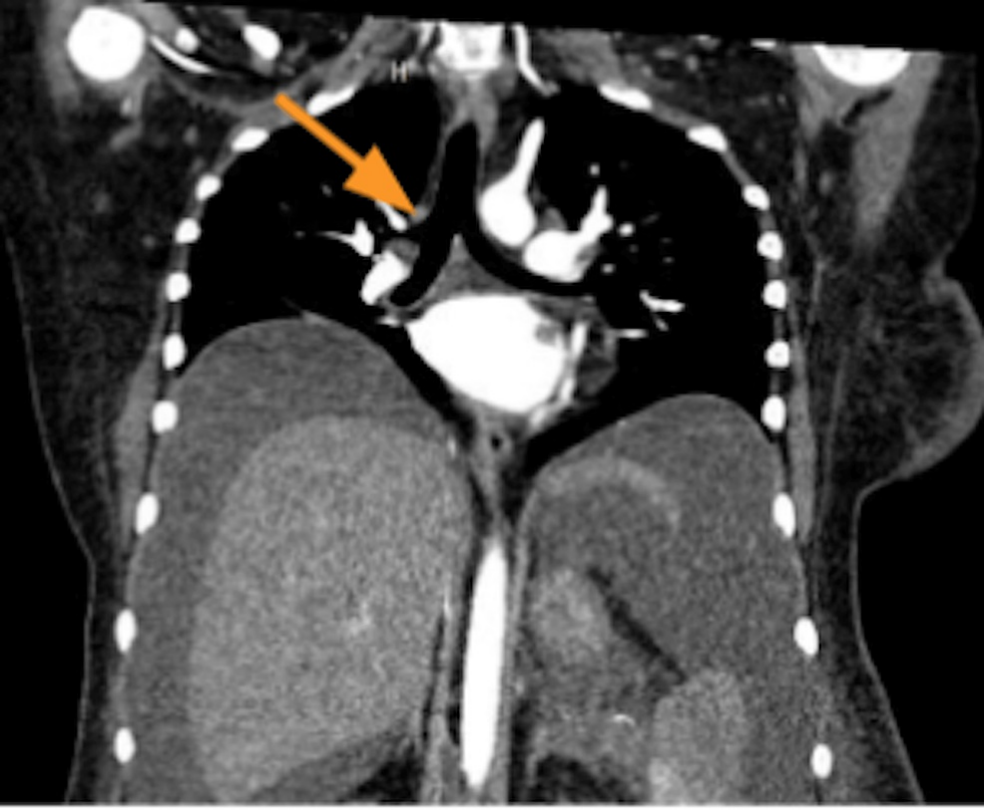
Pre-operative CT imaging of our patient shows tracheal bronchus. Intraoperative fiberoptic bronchoscopy confirmed the presence of an accessory right upper bronchus arising just above the carina almost like a trifurcation (shown with the arrow). This type of tracheal bronchus is also called “Bronchus Suis” or "Pig Bronchus".

Bronchus suis is a variant of tracheal bronchus, which supplies the entire upper lobe of the lung [1-4]. Three subtypes of tracheal bronchus are described in the literature according to the origin and location of the aberrant bronchus [1-3]. Type I is when tracheal bronchus arises more than 2 cm above the carina with distal tracheal narrowing [1-3]. It is the most severe form, manifesting with advanced symptoms of wheezing or stridor in early childhood [1-3]. Type 2 is when tracheal bronchus arises more than 2 cm above the carina, with no tracheal narrowing [1-3]. In type 3, the tracheal bronchus is located less than 2 cm above the carina, resembling trifurcation of the carina, which is similar to the one seen in our case (Figure 1) [1-3]. 

Rare forms of tracheal bronchus are: displaced (a single branch of the upper lobe bronchus arises above the carina); supernumerary (the right and left main bronchi branch out in normal anatomy but there is an additional bronchus arising above the carina); and rudimentary tracheal bronchus (blind-ended supernumerary bronchus) [2].

In adults, tracheal bronchus may present as recurrent upper or lower respiratory infections, mild asthma, bronchiectasis, and reduced exercise tolerance [1-4]. In children, narrowing or occlusion of this small aberrant bronchus leads to wheezing, air-trapping, atelectasis, and recurrent infections [1-5]. This may go undiagnosed or be initially considered to be asthma [2,5]. It is more likely in children with congenital abnormalities involving the respiratory and musculoskeletal systems e.g. Down syndrome, VATER syndrome, tracheoesophageal fistula, spinal fusion defects, or congenital cardiac defects [2,5].

Mild symptoms are treated with inhaled corticosteroids, bronchodilators, and antibiotics [2,5]. For patients unresponsive to medical management, surgical interventions such as tracheoplasty, segmentectomy, or lobectomy may be indicated to improve pulmonary function [2,5].

Prompt identification and definitive management are important for the prevention of adverse respiratory events. Interestingly, our patient’s preoperative CT depicted the tracheal bronchus (Figure 1) but was not mentioned in the original reading. Chest x-ray does not always produce a clear image for the identification of this anomaly. The gold-standard test for diagnosing tracheal bronchus and any of its variants is a multi-detector CT (MDCT) scan, which provides non-invasive 3D-reconstructive imaging of the tracheobronchial anomalies [2,5]. Fiberoptic bronchoscopy can also be used for the diagnosis of a tracheal bronchus [2.5].

For patients with known tracheal bronchus, it is essential to secure the ETT at the correct insertion distance and auscultate post-intubation to confirm bilateral breath sounds. The anesthesiologist should anticipate the potential for acute respiratory decompensation caused by the movement of ETT leading to obstruction of the tracheal bronchus opening [2-5]. A shorter ETT may also be considered to prevent entry into the tracheal bronchus [2,5]. If one-lung ventilation is necessary, isolation and ventilation of the lung may be challenging or even unsuccessful [2,4]. Alternatively, a bronchial blocker may provide effective lung isolation for procedures requiring one-lung ventilation [4]. 

## Conclusions

Tracheal bronchus and its variants are rare anomalies, mostly discovered incidentally. An anesthesiologist should consider the possibility of anatomical variations in the tracheobronchial tree during the management of airway emergencies. It is also crucial to identify and document the findings of these anomalies to alert and inform other physicians to prevent any complications should the patient have other procedures in the future.
